# Validating Test Score Interpretations Using Time Information

**DOI:** 10.3389/fpsyg.2019.01131

**Published:** 2019-05-28

**Authors:** Lena Engelhardt, Frank Goldhammer

**Affiliations:** ^1^DIPF – Leibniz Institute for Research and Information in Education, Frankfurt, Germany; ^2^Centre for International Student Assessment (ZIB), Frankfurt, Germany

**Keywords:** validity evidence based on response processes, speed, component skills, time on task effect, processing times

## Abstract

A validity approach is proposed that uses processing times to collect validity evidence for the construct interpretation of test scores. The rationale of the approach is based on current research of processing times and on classical validity approaches, providing validity evidence based on relationships with other variables. Within the new approach, convergent validity evidence is obtained if a component skill, that is expected to underlie the task solution process in the target construct, positively moderates the relationship between effective speed and effective ability in the corresponding target construct. Discriminant validity evidence is provided if a component skill, that is not expected to underlie the task solution process in the target construct, does indeed not moderate the speed-ability relation in this target construct. Using data from a study that follows up the German PIAAC sample, this approach was applied to reading competence, assessed with PIAAC literacy items, and to quantitative reasoning, assessed with Number Series. As expected from theory, the effect of speed on ability in the target construct was only moderated by the respective underlying component skill, that is, word meaning activation skill as an underlying component skill of reading competence, and perceptual speed as an underlying component skill of reasoning. Accordingly, no positive interactions were found for the component skill that should not underlie the task solution process, that is, word meaning activation for reasoning and perceptual speed for reading. Furthermore, the study shows the suitability of the proposed validation approach. The use of time information in association with task results brings construct validation closer to the actual response process than widely used correlations of test scores.

## Introduction

Assessing the validity of the intended test score interpretation is critical when drawing conclusions based on test scores. Various sources of validity evidence were described in the Standards for Educational and Psychological Testing (American Educational Research Association [AERA], American Psychological Association [APA], and National Council on Measurement in Education [NCME], 2014). One such source is evidence based on response processes. Information on the response process is now more easily available than ever before due to computer-based assessments and is also closer to the actual response process than test scores (Kane and Mislevy, 2017). For instance, information from the response process, namely processing times, has been used to support construct interpretation for mental rotation tasks: Spatial rotation theory postulates that mental rotation should proceed similarly to physical rotations. Indeed, the physical angle of the rotation object predicted not only item difficulty but also processing time (Bejar, 1990; Embretson, 1994). However, referring to the response process can be challenging if no single process model exists and various cognitive processes are involved in the task solution, as is the case for reading and reasoning tasks (Kane and Mislevy, 2017, p. 11).

The aim of this paper is to propose a construct validation approach that uses information from the response process, namely, processing times. This approach does not require complete process models, but simply assumptions about underlying component skills of the response process that are related to automation of information processing elements. These component skills have previously been used in classical approaches like the nomothetic span approach (Embretson, 1983), which investigates relations between test scores and other constructs as validity evidence (American Educational Research Association [AERA], American Psychological Association [APA], and National Council on Measurement in Education [NCME], 2014). Our proposed construct validation approach combines the nomothetic span approach with the relation of speed to ability.

Relations of speed to ability can be considered at different levels. The within-person level refers to the relation of effective speed to effective ability within a person, which can typically be investigated by observing a person completing a task under multiple experimental speed conditions (e.g., Goldhammer et al., 2017b). The obtained speed-ability relation is always negative as predicted by the speed-ability tradeoff (van der Linden, 2009). In contrast, our proposed validation approach is based on the speed-ability relationship at the between-person (or population) level. That is, persons complete a test without any speed manipulation. The observed speed-ability relation can be positive, zero, or negative depending on characteristics of the person and item level.

## Speed and Ability

Recent technologies offer the opportunity to record not just the product of task performance, that is the task solution, but also aspects of the behavioral process, for example, by recording time information or eye movements. Previous research using process data indicates that experts’ task solution process tends to differ from that of novices. Higher reading skills are associated with less and shorter fixations, longer saccades and fewer regressions (Rayner, 1998). Chess experts detect relevant information on a chess board faster, on average, than chess novices (Sheridan and Reingold, 2014). Chess experts were also four times faster than novices in a visual chess task and times for this task even correlated with the degree of expertise, measured as Elo ratings for experts or hours practicing chess per week for novice players (Sheridan and Reingold, 2017). In matrices tasks, total test scores were correlated with different task solution behaviors; they were positively related to the proportion of total time spent on inspecting matrices and negatively related to the proportion of total time spent on the response options (Vigneau et al., 2006). According to these studies, the task solution behavior of more proficient persons tends to differ across domains from that of less proficient persons, indicating differences in the cognitive processes underlying task solution.

Information about the time test-takers spend on each task is available by default nowadays in computer-based assessments. Time information carries information about the duration of the performed cognitive processes, with the limitation that the time a person spends on a given task might not only reflect task-related cognitive processes, but also non-task-related processes; for instance, it might also be affected by engagement (cf. response time effort; Wise and Kong, 2005). However, correct solutions do indicate a “successful mental process” (Hornke, 2000, p. 182), making it reasonable to interpret time as the duration of task-related cognitive processes, especially in the case of fast and correct responses. Also, rapid guessing may be associated with correct responses although not consistently and by chance, respectively. In this study, we interpret time information as the duration of the cognitive processes but consider processing times only in relation to the outcomes of this cognitive processing, namely response accuracy as an indicator of ability. Note that when referring to speed and speed-ability relationships, higher speed always means shorter time. If results are reported from studies in which response times were used, we have reversed the effects to also interpret them consistently in terms of speed.

### Relations Between Speed and Task Success at the Between-Person Level

The relation between speed in a task and the probability of task success is described as the ‘time on task effect’ (Goldhammer et al., 2014) and has been investigated in various studies with regard to item difficulty, person ability, and different domains (Goldhammer et al., 2014, 2015; Becker et al., 2016; Naumann and Goldhammer, 2017; see also Weeks et al., 2016). The time on task effects is modeled as the (average) effect of speed in an item on task success and the effect may vary across persons and items (fixed and random effect).

The average effect of speed in a task on the probability of a correct task solution has been found to be positive, zero, or negative in different studies. This means that in some assessments, more speed was associated with a higher probability of task solution, while in other assessments, less speed was associated with a higher probability of task solution. The direction of the relation depends *first* on the kind of cognitive processes required by a task. Goldhammer et al. (2014) reported that speed in a problem-solving task was associated with a lower probability of task solution, and for a reading task with a higher probability of task solution. The different directions were explained by differences in the task demands. Problem solving was assumed to require more controlled processing; thus, higher speed in a task was associated with a lower probability of a correct task solution. Reading was assumed to be based more on automatic processes; thus, higher speed in a task was associated with a higher probability of a correct task solution. Hence, the relation between speed in a task and the probability of a correct task solution was considered to depend on the cognitive processes performed in a task: whether they were more automatic or controlled.

The direction of the relation depends *secondly* on the interaction between person ability and item difficulty. Higher speed in a task is associated with a higher probability of task solution for more able persons working on rather easy items, and with a lower probability of task solution for less able persons working on harder items. Irrespective of whether the average effect of speed in a task on the probability of a correct task solution is positive, zero, or negative, across domains the effect varies consistently in that it is more positive, or less negative, for persons with higher abilities, compared to persons with lower abilities, and for easier items compared to harder items. Such variations have been found across domains, for instance in reading, problem solving, and reasoning (Goldhammer et al., 2014, 2015; Becker et al., 2016; Naumann and Goldhammer, 2017; see also Weeks et al., 2016; Bolsinova et al., 2017a; De Boeck et al., 2017; Chen et al., 2018). Thus, the relation between speed in a task and the probability of a correct task solution for a specific test in a certain domain can be positive for one group and negative for another. Different relations for speed in a task and the probability of a correct task solution indicate that the performed cognitive processes differ.

### Theoretical Models for Speed in a Task and Task Success

A number of different – possibly domain-specific – models explain why persons differ in their cognitive processes when they solve a task depending on their proficiency.

The distinction between the two kinds of cognitive processes explaining variation in the time-on-task effect stems from dual processing theory (Schneider and Shiffrin, 1977; Schneider and Chein, 2003): Automatic processes are well learned, run in parallel, and are unaffected by cognitive load. Controlled processes require attention, run serially, and depend on cognitive load. Controlled processes can also run automatically when they are well learned (cf. Ackerman, 1988). Persons who cannot solve tasks in automatic mode need to perform controlled processes, which leads to higher cognitive load and exceeds cognitive resources at some point (Sweller et al., 1998). Persons with a high proportion of automatized processes will solve items with high speed and high accuracy and solve even hard items correctly, because working memory can handle items with a higher cognitive load in the presence of more automatized processes. Persons with fewer automatized processes will need more time for correct solutions and will not be able to solve hard items correctly, because controlled processes are impaired by cognitive load.

Becker et al. (2016) also referred to cognitive processes performed in an automatic or controlled mode for matrices tasks. They stated that for very easy tasks, task complexity is low, which leads to a low cognitive load and automatic processing. In very hard tasks, task complexity is high, which leads to a high cognitive load and controlled processing. For items that are in between, more able respondents will be able to solve them in an automatic mode, while less able respondents will need to solve them in a controlled mode. If mental load is too high, working memory operates at its capacity limit increasing the probability that the task cannot be solved correctly.

Naumann and Goldhammer (2017) explained the difference between more and less proficient readers with reference to the compensatory-encoding model (Walczyk, 1995). This model posits that automatic reading processes on the word level are important for text comprehension. Readers with less automatized processes need to compensate for this deficit by performing these processes in a controlled mode. For less proficient readers, this might still lead to a correct, but slower, solution for relatively easy items and will burden working memory. As a consequence, as cognitive load increases, working memory will at some point reach its limit, meaning that the respondent will not be able to solve a task with a high cognitive load correctly (Sweller et al., 1998).

We assume that dual processing theory, extended by cognitive load theory (Sweller et al., 1998) as described by Becker et al. (2016), explains the relation between speed in a task and response accuracy in tasks where more than a single cognitive process is involved in task solution. Complex tasks like reading comprehension or reasoning (Kane and Mislevy, 2017) are based on numerous processes, and component skills enable the automation of information processing elements. Domain-specific theories explain which component skills are important for the automatization of tasks in a given domain. For instance, the automatic lexical access to word meaning is essential for reading comprehension (cf. Perfetti, 2007).

The proposed validation approach is suitable for tasks following the dual processing theory classification. There might be tasks for which this assumption does not hold, for example tasks which mainly require knowledge, in which domain-specific component skills might not be involved in the task solution process and automatization does not provide any advantage. In some tasks, other factors like decision-making speed might also matter, and models other than the dual processing framework are more suitable for describing the relation between task speed and accuracy. For example, diffusion models are typically used for very easy two-choice response tasks requiring short response times. Such models represent the response process as an information accumulation process the proceeds until enough evidence for one of the two choices is collected (van der Maas et al., 2011).

## Validity Approach Using Time Information

Although validating test score interpretations based on response processes (cf. American Educational Research Association [AERA], American Psychological Association [APA], and National Council on Measurement in Education [NCME], 2014) is closer to the actual cognitive processes than merely using assessment results, providing this kind of validity evidence can be challenging if no single process model is available (Kane and Mislevy, 2017), as is the case for reading tasks (Kintsch, 1998), for instance. The following validation approach allows for investigating the validity of test score interpretations from assessments of complex constructs such as reading comprehension by using processing times as generic information about the response process.

The validation approach is based on the latent effect of the person variable speed on ability. Following van der Linden (2007), speed in a task (i.e., processing time observed for a task) depends on a person-specific and an item-specific component. The person-specific component (effective) speed represents inter-individual differences in time use, and it is assumed to be the same across all items (although the weighting can vary across items, see Klein Entink et al., 2009). The item-specific component describes an item’s time intensity and the difference between the observed processing time and the expected processing time given the person-/item-specific components represents the residual. Just as ability is estimated based on all item responses, the speed is estimated according to time use across all items.

The effect of speed on ability is assumed to be more positive (or less negative) in a group of strong test-takers and vice versa. This kind of moderation has been shown by previous empirical studies which revealed differences in the relationship between speed (e.g., average item response time or time for a specific processing behavior) and ability (e.g., test score) depending on item difficulty or person ability (Neubauer, 1990; Knorr and Neubauer, 1996; Rayner, 1998; Vigneau et al., 2006; Sheridan and Reingold, 2014, 2017).

**FIGURE 1:**
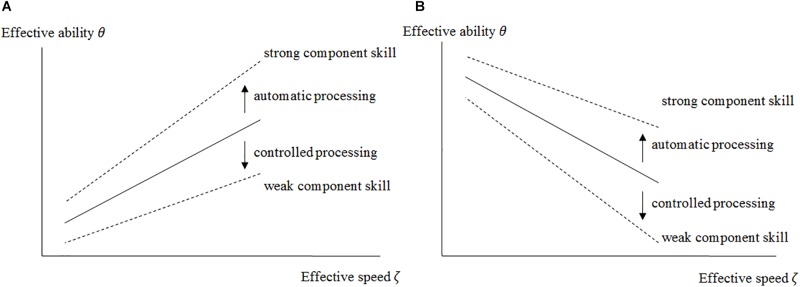
Illustration of the relation of effective speed to effective ability at the between person level (**A:** positive relation, **B:** negative relation). The upper/lower dashed line indicates how a strong/weak construct-related component skill is expected to moderate the speed-ability relation. For persons with strong component skill it is expected to be more positive and less negative, respectively (and vice versa).

We assume that in complex tasks, differences in the speed-ability relation depend on automatized sub-processes, and thus on well-developed component skills. Persons with better component skills will be able to perform sub-processes in an automatized mode, while persons with weaker component skills will perform these sub-processes in a controlled mode (cf. *dual processing theory*; Schneider and Shiffrin, 1977; Schneider and Chein, 2003). The automatized mode enables fast correct solutions. In contrast, the controlled mode allows correct but slower task solutions and is affected by a high cognitive load. Consequently, solving tasks with a high cognitive load in a controlled mode will exceed working memory capacity at some point (Sweller et al., 1998), and prevent the respondent from solving these tasks successfully. Based on the dual-processing theory together with cognitive load theory it is expected that the strength of the component skill has an impact on the observed speed-ability relation. If these differences in the speed-ability relation depend on certain component skills predicted by domain-specific theories, the relation between speed and ability in a sample would be positively moderated by these component skills. In turn, *a component skill is involved in a task’s response process and supports a fast and correct task solution process if it positively affects the relation between speed and ability in the target domain* (cf. Figure 1). *Such a positive interaction effect supports the validity of the construct interpretation assuming that a task’s solution process requires these component skills.*

The presented validation approach focuses on person-level variables. It is based on the assumption that differences in the relation between effective speed and effective ability describe differences in cognitive processes. Person-level variables, for example component skills that theoretically underlie the task solution process, are assumed to refer to those differences in cognitive processes and should hence moderate the speed-ability relation.

### Interpretations of Moderation Effects

Before the moderation effects are discussed, the main effects of effective speed are focused. *A* *positive* *main effect of effective speed on the effective ability* (cf. Figure 1A) means that persons with a higher effective speed show also a higher effective ability. This positive effect suggests that processes amenable to automation were involved in performing these tasks and that, in persons working both fast and successfully, these processes are automatized to a large extent. Lower speed and lower ability would result for those persons who compensate non-automated processes by controlled processes. While for simple tasks controlled processing would lead to correct and slower task solutions, difficult tasks would be wrong due to limited working memory capacity. *A negative main effect of effective speed on the effective ability* (cf. Figure 1B) means that persons with a higher effective speed have a lower effective ability and vice versa. This suggests that controlled processes were more likely to be used to perform these tasks. A lower speed along with higher ability would result for those who performed these tasks in a controlled mode. For those who did not thoroughly engage into the solution process and/or gave up at an early stage, this would result in higher speed and lower performance at the same time. Such a disengaging behavior could be driven by individual expectations of task success (cf. intentional omissions, Mislevy and Wu, 1996; see also Goldhammer et al., 2017a). Thus, potential sources of variations in effective speed may originate not only from the performed task-specific cognitive processes but also from the test-taker’s meta-cognition about performing the task.

*A positive moderation effect* of a component skill on the speed-ability relationship means that the speed-ability relation becomes more positive or less negative. The positive moderation of the positive speed effect (see Figure 1A) indicates that for persons being in command of well-automatized procedures (e.g., lexical access), effective speed (e.g., in reading) more strongly reflects individual differences in ability (e.g., reading comprehension). For persons with weak component skills, however, effective speed is less (or even negatively) related to ability, since time-consuming controlled or strategic processes have to be performed to obtain a correct response. The positive moderation of the negative speed effect (see Figure 1B) indicates that for persons with strong component skills effective ability would be less impaired in the situation of high effective speed; put differently, such persons may afford to work fast to some extent given highly automatized elements of cognitive processing. The opposite is true for persons with weak component skills. Here, the detrimental effect of fast controlled processing is strengthened. A positive moderation effect supports that the respective component skill is associated with automatic processing in the target domain and would thus provide convergent validity evidence for the construct interpretation of the ability test score, if this component skill is theoretically assumed to underlie the task solution process. Conversely, a component skill that underlies the task solution process according to an alternative theory (cf. Kane, 2013), but does *not* moderate the speed-ability relation in a positive direction, would support the intended theory-based interpretation and provide discriminant validity evidence for the construct interpretation of the test score.

*A negative moderation effect* of a component skill on the speed-ability relationship would indicate that the difference in effective ability between persons with strong vs. weak component skills becomes even smaller for persons with higher effective speed (the lines in Figure 1 would converge at high effective speeds). As described, if a component skill was responsible for performing processes in automated mode, the difference should be higher at higher speed. A negative moderation effect would therefore support that this component skill was *not* associated with automatic processing in the target domain. Instead, the advantage for persons with strong component skills in the event of a negative moderation effect is greater for those who worked slowly. In the case of a negative main effect of speed, for example, the component skill could be a resource that contributes to the correct solution of the task, particularly when working at low levels of speed.

### Assumptions

Please note that the conclusions that are drawn from these moderation effects are different from those that can be drawn based on the pure main effects of component skills on target ability: The main effects of component skills on target ability describe the relationships between two ability variables being defined by item response variables (i.e., correctness of task results). The moderation effects, however, consider not only the task outcomes but also the speed at which these results were achieved, that is, the process of task completion. Consequently, a failure to find a main effect would mean that persons with high component skills do not reach higher test scores in the target ability. A failure to find a moderation effect would mean that persons with higher component skills do not show a more positive relation of ability and speed in the target construct, hence, that this component skill is not related to underlying automated processes. The time a person takes on an item also depends – especially when the item is solved incorrectly – on motivational factors like the willingness to perform the tasks as instructed (cf. test-taking effort; Wise and DeMars, 2005). This means that a respondent who takes a relatively short time across items can be indicative of a high degree of automated processes, but also low engagement throughout the test (cf. Goldhammer et al., 2017a). However, only persons with automated processes will be able to solve tasks in a domain correctly and with high speed. Hence, we assume that considering speed across tasks, together with ability, allows for interpreting differences in the relation between speed and ability in terms of differences in cognitive processing. Still, this approach depends on the assumption that most persons perform task-related processes. If many persons do not behave as intended, it will be hard to detect moderating variables for the speed-ability relationship. This can be especially problematic when many respondents perform rapid guessing.

Just as in other correlational approaches, person variables might moderate the speed-ability relation not because they are part of the assumed task solution process but because they correlate with other third variables that describe why persons work faster and rather correctly. It clearly follows from this that the construct validity of the interpretation of the component skill score is a crucial precondition for the suggested approach.

Furthermore, the extent to which the target ability and the component skill overlap is crucial for our approach: The component skill should describe a relevant sub-process of the cognitive processes in the target construct that can be automated. If the component skill represents only a rather irrelevant aspect of the cognitive processes in the target ability, performing this process automatically will not significantly moderate the speed-ability relationship in the target construct. If the component skill represents the cognitive processes in the target ability to a very large extent, the component skill would strongly predict the target ability, making an interaction effect between target speed and component skill unlikely. Furthermore, our approach is based on differences in cognitive processes as represented by the speed-ability relation in the target construct and in the respective component skills. Therefore, the sample needs to include such differences. Heterogeneous samples, such as those in the PIAAC study, are likely to meet these preconditions.

### Classification of the Validity Approach

In terms of classical validation approaches, the proposed validation approach of examining relations with component skills can be seen as similar to the nomothetic span approach (Embretson, 1983) or collecting validity evidence based on relations with other variables (American Educational Research Association [AERA], American Psychological Association [APA], and National Council on Measurement in Education [NCME], 2014). However, our approach does not focus on relating test scores of the target construct (e.g., reading) and component skills (e.g., lexical access) to each other. Instead, we analyze whether component skills moderate the relation between speed and ability. If a component skill that is theoretically assumed to be elicited by the task actually moderates the relation between speed and ability (cf. Figure 1), it supports the notion that this component skill is indeed involved in the response process of this task. Consequently, such a result provides validity evidence for the construct interpretation of the test scores based on response processes (American Educational Research Association [AERA], American Psychological Association [APA], and National Council on Measurement in Education [NCME], 2014). The nomothetic span approach focuses on individual differences rather than on differences between items (cf. *construct representation* *approach*; Embretson, 1983). This is also why our suggested approach focuses on the person-specific time component that underlies response times in all items (van der Linden, 2007) rather than on the time a person takes on single items.

## Research Questions

The overall empirical goal of this study is to test the proposed validity approach based on processing times. Two cognitive constructs, reading comprehension and reasoning, were selected to investigate the validity of the construct interpretation of related test scores. The literacy competence test from the Program for the International Assessment of Adult Competencies (PIAAC; OECD, 2016) was used for assessing reading comprehension and the Number Series Test (McArdle and Woodcock, 2009) for assessing reasoning. Competencies such as reading comprehension are assumed to matter for the handling of very specific situations, whereas general cognitive skills can be applied to a wide range of situations (Klieme et al., 2008).

The following sections describe component skills underlying reading and reasoning that are thus critical for automated processing. They are assumed to moderate the speed-ability relationship in a positive direction. A positive moderation by the component skill that theoretically underlies the task solution process would provide convergent validity evidence. Kane (2013) argues that test score interpretations should be put to the test. In traditional approaches, such as analyzing relations to other variables (e.g., American Educational Research Association [AERA], American Psychological Association [APA], and National Council on Measurement in Education [NCME], 2014), this type of challenging analysis would be done to provide discriminant validity evidence for the construct interpretation. In a similar vein, in our approach, discriminant validity evidence would be provided if a component skill that is believed to underlie the task solution process according to alternative theories does not moderate the relation between speed and ability in a positive direction.

### Reading

Literacy items in PIAAC are assumed to involve “a range of skills from the decoding of written words and sentences to the comprehension, interpretation, and evaluation of complex texts” (OECD, 2016, p. 18). Kintsch (1998) describes reading as the interplay of bottom-up and top-down processes in his construction-integration model. Bottom-up processes are performed to process words in order to build a propositional representation of the text. Then, knowledge is integrated in top-down processes to construct a situation model. One bottom-up-process that is theoretically involved in reading (Kintsch, 1998) and also an empirical predictor of reading comprehension (Perfetti, 2007) is the activation of word meanings. Word reading is a process that can be automatically performed (Augustinova and Ferrand, 2014). Thus, the relationship between speed and ability in reading is assumed to be influenced by the extent to which readers activate word meanings from the text in an automatic or controlled mode and should be more positive for automatic activation. Knowing more words might prevent a person from encoding letters separately or from guessing the meaning from the context. In the context of cognitive load (Sweller et al., 1998), not knowing words might burden working memory capacity and might not only prevent faster task solution in easy items but might even more so prevent correct task solution on harder items.

If the relation between speed and ability in reading is more positive among persons with greater word meaning activation, this provides convergent validity evidence for the construct interpretation, because it indicates that the solution process in reading tasks requires reading-specific component skills (Hypothesis 1a).

The wide range of situations to which general cognitive skills can be applied bring them into play as an alternative interpretation of competence scores. Whether competence tests used in large-scale assessments are also based on general cognitive skills and to what extent they represent the outcomes of learning processes have been investigated in numerous studies based on item scores (Brunner, 2005; Nagy, 2006; Rindermann, 2006; Prenzel et al., 2007; Baumert et al., 2009; Rindermann and Baumeister, 2015; Saß et al., 2017). The construct interpretation should be challenged through alternative interpretations that see general cognitive skills as also involved in literacy items. An important component skill of general cognitive skills is perceptual speed (e.g., Vernon et al., 1985).

If the relation between speed and ability in reading is not more positive among persons with higher perceptual speed, this provides discriminant validity evidence for the construct interpretation, as it indicates that the solution process in reading tasks does not involve reasoning-specific component skills (Hypothesis 1b).

### Reasoning

Fluid reasoning is assumed to be a good indicator for general cognitive skills (Vernon, 1965). Reasoning requires controlled mental operations to solve novel problems. Deductive/inductive reasoning and quantitative reasoning are considered to belong to the broad category of fluid reasoning alongside other constructs. Fluid reasoning is required to accomplish cognitively complex tasks and is hence based on various elementary cognitive processes (McGrew, 2009). The elementary cognitive processes underlying reasoning processes include working memory capacity and perceptual speed (Vernon et al., 1985; Neubauer, 1990; Schweizer and Koch, 2002; Altmeyer et al., 2009). Perceptual speed describes the ability to perform easy and elementary cognitive tasks automatically, and is one of the specific, narrower abilities involved in processing speed (McGrew, 2009). Higher perceptual speed can lead to faster solutions on easy fluid reasoning tasks and correct solutions on demanding tasks, because it allows a greater amount of information to be processed despite limited working memory capacity. Slow processes, in contrast, may lead to a loss of information and a slow or even incorrect task solution (Jensen, 1982; Vernon et al., 1985; Sweller et al., 1998).

If the relation between speed and ability in reasoning is more positive among persons with greater perceptual speed, this provides convergent validity evidence for the construct interpretation, as it indicates that the solution process in reasoning tasks requires reasoning-specific component skills (Hypothesis 2a).

Although it is has been shown that schooling can affect reasoning (Ceci and Williams, 1997; Guill et al., 2017), we assume that highly specific component skills of education-related competencies, such as word meaning activation, do not moderate the relation between reasoning speed and reasoning ability in a positive direction.

If the relation between speed and ability in reasoning is not more positive among persons with higher word meaning activation, this provides discriminant validity evidence for the construct interpretation, as it indicates that the solution process in reasoning tasks does not involve component skills that are related to reading (Hypothesis 2b).

## Materials and Methods

### Sample

This study is based on data from the PIAAC-L study (GESIS – Leibniz Institute for the Social Sciences, German Socio-Economic Panel (SOEP) at DIW Berlin and LIFBI – Leibniz Institute for Educational Trajectories, 2017; Rammstedt et al., 2017). In PIAAC-L, all German respondents from the PIAAC study were re-contacted in 2015 and received either (randomly selected) PIAAC literacy items (*N* = 1423) or other instruments. One year later, respondents from the 2015 assessment were re-contacted again and all received measures from the Socio-Economic Panel (SOEP; Schupp et al., 2008), the Symbol-Digit Test (Schupp et al., 2008) and a multiple-choice vocabulary intelligence test (Lehrl, 2005). Some of those respondents were also selected to complete the Number Series Test in 2016 (McArdle and Woodcock, 2009; Engelhardt and Goldhammer, 2018) based on the instruments they had received in 2015. The data set used for the analyses in this study consists of *N* = 1588 respondents. Of those, *N* = 744 respondents completed the PIAAC literacy items and the Number Series, *N* = 679 only the PIAAC literacy items, and *N* = 165 only the Number Series. In the whole data set, respondents were *M* = 42.41 years old (*SD* = 13.72; Min = 19, Max = 69) on average in 2015, and 48.55% were male (51.45% female).

### Measures

The PIAAC literacy test^1^ included a total of 49 dichotomously scored items and is assumed to assess reading competence (cf. OECD, 2016). PIAAC is an OECD study that aims to assess adults’ competencies in literacy, numeracy, and problem-solving in an international comparison. These “key information-processing competencies” (OECD, 2016, p. 16) are necessary, for example, to participate in social life or the labor market. In addition, they are also assumed to be transferable to different situations and learnable.

**FIGURE 2:**
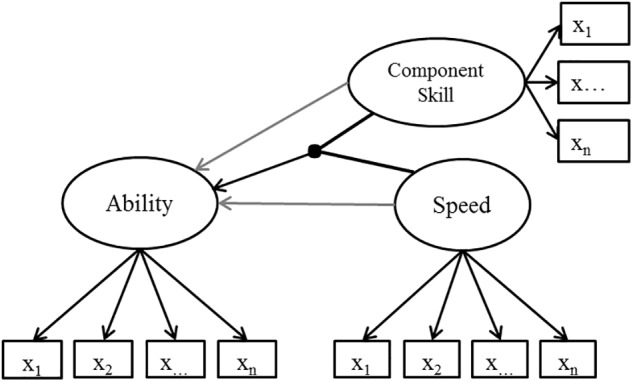
Structural model to investigate how the component skills [perceptual speed (PS) and word meaning activation (WMA)] moderate the relation between speed and ability in reading and reasoning tests (latent correlations are omitted).

In PIAAC, literacy is defined as “understanding, evaluating, using and engaging with written texts to participate in society, to achieve one’s goals, and to develop one’s knowledge and potential” (OECD, 2016, p. 19). The reading tasks can contain a continuous text, a non-continuous text (e.g., form), or both, and can even contain more than one text. Each reading task also requires one of three cognitive strategies (access and identify, integrate and interpret, or evaluate and reflect), and can address topics related to work, personal matters, society and community, or education and training. The example item (see footnote 1) “preschool rules” requires the cognitive strategy “access and identify” and takes up a personal topic. In this item, test-takers have to take, from a text, by what time, at the latest, children have to arrive at preschool. The text contains nine bullet points from one or two short sentences and each bullet point describes a rule. Two of these rules contain time information, which makes it necessary to also read the text in which the time information is embedded in order to solve the item. Regardless of the type of text (continuous texts, non-continuous texts, mixed texts, or multiple texts), cognitive strategy (access and identify, integrate and interpret, or evaluate and reflect), or topic (work-related, personal, community and society, or education and training), test takers must comprehend text in each PIAAC literacy item. It is assumed that word activation skills support the task solution in terms of speed and accuracy, as the fast retrieval of word meaning from memory supports the correct semantic integration of words and, in turn, the comprehension of text. In addition, the automatic retrieval of words from memory reduces cognitive load and does not compromise the cognitive processes that are required for task solution. For this reason, across all items, word meaning activation skill is expected to moderate the relationship between speed and ability in reading. The literacy test was administered in a two-stage adaptive test design (Kirsch and Yamamoto, 2013, p. 10), with 9 out of 18 items administered in the first items and 11 out of 31 items administered in the second stage (OECD, 2016). The assessment had no time restriction. Respondents had an increased probability of receiving a testlet appropriate for their skill level depending on three variables (education level, native speaker, passing score on computer-based assessment core tasks; for more details, see OECD, 2016). Two separate latent factors were modeled on the basis of the literacy items: reading ability and reading speed. Fitting 2-parameter IRT models in Mplus (Muthén and Muthén, 2015) based on *N* = 1423 respondents revealed that all literacy item response variables loaded significantly on a joint latent ability factor [standardized loadings (variance of the latent variable fixed to one): *M* = 0.60; *SD* = 0.11; Min = 0.37; Max = 0.82; see the Appendix for further information]. Note that the MLR estimator (maximum likelihood estimation with robust standard errors), which was used to deal with the missing data structure, does not provide absolute model fit information. We refrain from presenting additional information on item fit given that both the PIAAC literacy test and the Number Series Test are well-established and trialed instruments. The reading speed factor was obtained as follows: item-level processing times, that is the total time a person spent on an item including editing or reviewing their answer, were at first log-transformed and then subjected to a confirmatory factor analysis. On average, respondents spent *M* = 72.87 s on an item (*SD* = 28.66; Min = 25.59; Max = 129.04). The log-transformed processing times for all items loaded on a joint latent factor (cf. Figure 2, which describes the model for data analyses) representing person-specific time use (standardized loadings: *M* = 0.59; *SD* = 0.07; Min = 0.46; Max = 0.75; see the Appendix for further information). For easier interpretation in terms of reading speed, we switched the polarity of the processing time results (from positive to negative and vice versa), such that higher values indicate greater speed and thus less time spent on an item. The model fit of the measurement model for the processing times was acceptable (CFI = 0.879; TLI = 0.872; RMSEA = 0.029; SRMR = 0.072).

Fifteen dichotomously scored Number Series items (McArdle and Woodcock, 2009) were used to measure fluid reasoning ability, because quantitative reasoning is a specific ability within the broader domain of fluid reasoning (McGrew, 2009). Each of the fifteen number series consisted of between four and seven numbers. One number was missing in each of the first 14 number series. Only in the fifteenth number series were two numbers missing. The missing numbers were either located at the beginning, in a middle position, or at the end of the number series. After 16 min, test-takers were navigated not to the next number series but to the end of the assessment (cf. Engelhardt and Goldhammer, 2018). This only happened to seven respondents. Two separate latent factors were modeled on the basis of the number series items: reasoning ability and reasoning speed. A 2-parameter IRT model was fitted, with all items loading significantly on a joint latent ability factor [standardized loadings (variance of the latent variable fixed to one): *M* = 0.69; *SD* = 0.11; Min = 0.52; Max = 0.87; *N* = 909 respondents; see the Appendix for further information]. In order to test the measurement model for a joint latent reasoning speed factor, the total processing times, that is, the time in seconds a person spent on a single item including editing or reviewing their answer (*M* = 28.40; *SD* = 26.33; Min = 5.36; Max = 110.61), were first log-transformed. Then, a confirmatory factor analysis of the log-transformed processing times was conducted to model a latent factor (cf. Figure 2) representing person-specific time use. The polarity of the processing time results was changed for the results presented in the tables. That is, higher values indicate less time spent on an item (higher speed). Due to an unacceptable model fit (CFI = 0.707; TLI = 0.658; RMSEA = 0.129; SRMR = 0.103), correlations between items were allowed back into the model one-by-one according to the modification indices until the model fit reached an acceptable level (CFI = 0.966; TLI = 0.948; RMSEA = 0.050; SRMR = 0.043). Correlations were only added between very easy items and between hard items. This indicates that not only did persons differ in their general reasoning speed, but that there were also differential differences in reasoning speed for easy and harder items. In the final model, all items loaded on the reasoning speed factor (standardized loadings: *M* = 0.51; *SD* = 0.15; Min = 0.16; Max = 0.68; see the Appendix for further information).

For the two component skills, word meaning activation and perceptual speed, one latent factor was modeled per test. Perceptual speed is frequently assessed using the Symbol-Digit Test^2^ (Ackerman, 1988). This test was also part of the Socio-Economic Panel (Schupp et al., 2008) and was thus used in this study. To complete the test, respondents recoded symbols into digits according to a legend. The legend consisted of nine symbols corresponding to the digits one to nine. Participants had 90 s to recode as many symbols as possible one after the other on the computer. Of these 90 s, the total number of correctly coded digits was recorded for three time intervals of 30 s each, known as parcels. A latent perceptual speed factor was modeled on the basis of these three parcels, leading to a fully saturated and therefore perfectly fitting model (see the Appendix for further information).

The Multiple-Choice Vocabulary Intelligence Test^3^ (Lehrl, 2005) from the Socio-Economic Panel (Schupp et al., 2008) was used to measure a component skill specific to reading. This test requires respondents to identify the existing word in 37 word groups of increasing difficulty. Each group consists of five potential German words, four of which are fictitious. Task completion time was not restricted (Zabal et al., 2016). Kintsch (1998) describes this skill as word meaning activation. Because we wanted subsequent analyses to be based on relations among latent variables, a single indicator model was used to estimate a latent variable for word meaning activation. In order to ensure model identification, the single indicator variable (i.e., the number of correct answers across all 37 items) was standardized, the variance of the latent factor was fixed to 1, and the factor loading was fixed to the root of 0.76 (see the Appendix for further information). This loading served as a proxy for the estimated reliability of this test and is based on its correlation with a similar test (Satzger et al., 2002).

### Data Analyses

Data was analyzed using Mplus (Muthén and Muthén, 2015). As the literacy items were administered in an adaptive design, the three context variables involved in testlet selection (education level, native speaker, passing score on computer-based assessment core tasks) were included as correlated variables for Hypotheses 1a and 1b in order to make it justifiable to assume that the not-administered items were missing at random (MAR; cf. Enders, 2010). The MLR estimator (maximum likelihood estimation with robust standard error) can be used to test structural equation models using categorical items. It also has the advantage of being able to consider all information under the missing at random (MAR) assumption despite the presence of missing data, making it suitable for the present study. Structural equation models for each domain were tested to analyze the hypotheses. First, only main effects for reasoning/reading speed were modeled as predictors of reading/reasoning ability (baseline model). To test the hypotheses, latent interaction terms (cf. latent moderated structural equations; Klein and Moosbrugger, 2000) of reasoning/reading speed and perceptual speed and of reasoning/reading speed and word meaning activation were included in the model for literacy (Hypotheses 1a and 1b) and number series (Hypotheses 2a and 2b). These models also contained the main effects of perceptual speed and word meaning activation. The model for the hypotheses is visualized in Figure 2.

## Results

To test the hypotheses, we analyzed whether the relation between speed and ability was positively moderated only by the domain-specific component skill, meaning that the relation between speed and ability is assumed to be more positive for persons with higher domain-specific component skills. The results are presented separately for reading (Table 1) and reasoning (Table 2).

**Table 1:** Interaction effects of component skills – word meaning activation (WMA) and perceptual speed (PS) – and reading speed on reading ability.

	Baseline model				Hypothesis 1a (convergent)				Hypothesis 1b (discriminant)			
	β	*SE*	*z*	*p*	β	*SE*	*z*	*p*	β	*SE*	*z*	*p*
Reading speed	–0.08^1^	0.04	–1.94^1^	0.053	–0.04^1^	0.04	–1.02^1^	0.310	–0.28^1^	0.04	–7.18^1^	<0.001
WMA					0.49	0.05	10.41	<0.001				
WMA × Reading speed					0.12^1^	0.03	3.89^1^	<0.001				
PS									0.49	0.03	16.32	<0.001
PS × Reading speed									–0.07^1^	0.03	–2.27^1^	0.023


*Baseline model: *N* = 1423; Hypotheses 1a and 1b: *N* = 1587. ^*1*^Data analysis was based on processing times (higher values indicating more time on an item). In order to interpret the obtained latent variables as “speed” (higher values indicating less time on an item), we inverted the polarity of the β- and *z-*values for speed in the table.*

**Table 2:** Interaction effects of component skills – word meaning activation (WMA) and perceptual speed (PS) – and reasoning speed on reasoning ability.

	Baseline model				Hypothesis 2a (convergent)				Hypothesis 2b (discriminant)			
	β	*SE*	*z*	*p*	β	*SE*	*z*	*p*	β	*SE*	*z*	*p*
Reasoning speed	0.27^1^	0.04	6.16^1^	<0.001	0.11^1^	0.05	2.17^1^	0.030	0.24^1^	0.04	6.15^1^	<0.001
WMA									0.46	0.04	10.78	<0.001
WMA × Reasoning speed									0.06^1^	0.04	1.43^1^	0.152
PS					0.35	0.04	8.36	<0.001				
PS × Reasoning speed					0.10^1^	0.04	2.50^1^	0.012				


*Baseline model: *N* = 909; Hypotheses 2a and 2b: *N* = 1410. ^*1*^Data analysis was based on processing times (higher values indicating more time on an item). In order to interpret the obtained latent variables as “speed” (higher values indicating less time on an item), we inverted the polarity of the β- and *z*-values for speed in the table.*

### Reading

Reading speed and reading ability were not significantly related in the sample (Table 1: β = -0.08, *p* = 0.053). When including perceptual speed in the analyses (cf. Results for Hypothesis 1b), the relation between reading speed and reading ability became more negative (Table 1: β = -0.28, *p* < 0.001). This might be because reading speed and the component skill perceptual speed were positively correlated (*r* = 0.37; *p* < 0.001).

**FIGURE 3:**
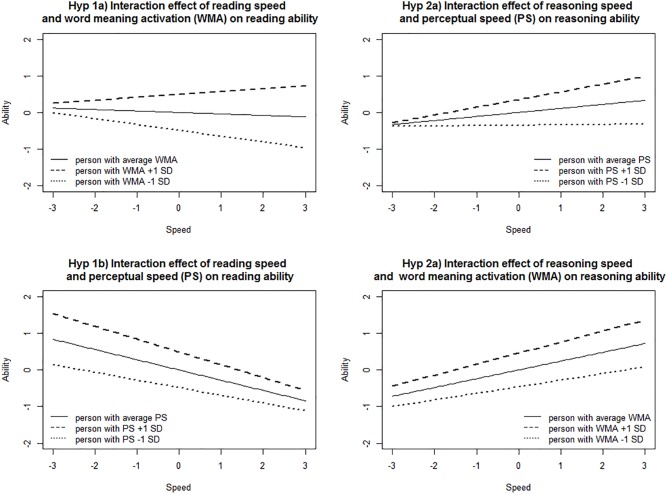
Moderation effects of perceptual speed and word meaning activation for the relation between speed and ability in the reading and reasoning tests.

Word meaning activation, as a domain-specific component skill, was positively associated with reading ability (main effect: β = 0.49, *p <* 0.001). As expected, the relation between reading speed and reading ability was positively moderated by word meaning activation (Hypothesis 1a; interaction effect: β = 0.12, *p* = 0.001). This indicates that persons with higher word meaning activation skills had higher reading ability scores (main effect) and those who worked faster had, in addition, higher reading ability scores, compared to those with lower word meaning activation skills (interaction effect; see Figure 3, Hypothesis 1a), because the relation between reading speed and reading ability was more positive for these persons, providing convergent validity evidence that word meaning activation is important for automated reading processes.

Perceptual speed, as a component skill of fluid reasoning, was also positively associated with reading ability (main effect: β = 0.49, *p <* 0.001). As expected, perceptual speed did not moderate the relation between reading speed and reading ability in a positive direction (Hypothesis 1b; interaction effect: β = -0.07, *p* = 0.023), but did so in negative direction. This indicates that persons with higher perceptual speed had higher reading ability scores (main effect) compared to persons with lower perceptual speed. But this advantage of having higher perceptual speed was smaller for those who worked faster (interaction effect, see Figure 3, Hypothesis 1b). The fact that no positive moderation effect was found provides discriminant validity evidence. Further interpretations of the negative interaction effect are presented in the discussion section.

### Reasoning

Reasoning speed and reasoning ability were positively related (Table 2: β = 0.27, *p <* 0.001) in this sample. The component skill perceptual speed was positively correlated with reasoning speed (*r* = 0.48; *p* < 0.001), which led to a less positive but still significant relation between reasoning speed and reasoning ability (Table 2: β = 0.11, *p =* 0.030) when the component skill perceptual speed was included in the model.

As expected, perceptual speed, a domain-specific component skill, was positively related to reasoning ability (main effect: β = 0.35, *p <* 0.001) and moderated the relationship between reasoning speed and reasoning ability in a positive direction (Hypothesis 2a; interaction effect: β = 0.10, *p =* 0.012). This indicates that persons with higher perceptual speed had higher reasoning ability scores (main effect) and those who worked faster had, in addition, higher reasoning ability scores compared to those with lower perceptual speed for (interaction effect; see Figure 3, Hypothesis 2a), because the relation between reasoning speed and reasoning ability was more positive for these persons. This provides convergent validity evidence that the component skill of perceptual speed is important for automated processes in reasoning tasks.

Word meaning activation was also positively associated with reasoning ability (main effect: β = 0.46, *p <* 0.001), but did not positively moderate the relation between reasoning speed and reasoning ability (Hypothesis 2b; interaction effect: β = 0.06, *p =* 0.152), providing discriminant validity evidence. This suggests that persons with higher word meaning activation skills had higher reasoning ability scores but this difference did not increase for those who worked faster (interaction effect; see Figure 3, Hypothesis 2b). Word meaning activation was as expected not important for automated cognitive processes in reasoning tasks.

## Discussion

### Main Findings

The results provided both convergent and discriminant validity evidence for the construct interpretation of reasoning and reading ability scores. Convergent evidence was provided because the relations between speed and ability were more positive among persons with stronger domain-specific component skills (word meaning activation for reading and perceptual speed for reasoning). This means that people with stronger component skills that were theoretically assumed to be relevant for the target ability did indeed obtain higher ability scores and this advantage was even more explicit when they worked faster, which supports that the component skills were indeed involved in automated task solution processes. Discriminant validity evidence was provided because the component skills that were assumed to be irrelevant for automated task solution processes in each domain did not moderate the speed-ability relation in a positive direction. Persons with higher scores on the irrelevant component skills and who worked faster did not show relative higher ability compared to persons with lower scores on the irrelevant component skills.

### Interpretation of Empirical Findings

Although empirical support was found for the hypothesized moderation effects, the two component skills (word meaning activation and perceptual speed) were positively associated with both abilities examined (reading and reasoning). One could ask whether these correlations between test scores for one ability and component skills for the other ability call the validity of the intended construct interpretation into question. We argue that this is not the case, because competencies and general cognitive skills are assumed to be related, for instance because schooling may affect reasoning, and there is ongoing discussion about the extent to which those skills can be separated (Brunner, 2005; Nagy, 2006; Rindermann, 2006; Prenzel et al., 2007; Baumert et al., 2009; Rindermann and Baumeister, 2015; Saß et al., 2017). Hence, it is not surprising that component skills for reading correlate with tests scores for reasoning tasks and vice versa, and this does not call the validity of the test score interpretation into question. Perceptual speed is considered to be a general and domain-unspecific skill. According to cognitive load theory (Sweller et al., 1998), fast processing might reduce cognitive load in complex tasks, which could in turn help with task solution even if a task’s cognitive load is high. Hence, persons with higher perceptual speed might also have advantages in tasks from other domains. Moreover, according to Cattell’s (1963) investment theory, fluid intelligence (e.g., reasoning) is important for the acquisition of crystallized abilities (e.g., reading). The reverse is also posited: Educational processes are assumed to affect fluid intelligence (Ceci and Williams, 1997; Guill et al., 2017). Hence, such correlations can actually be expected on the basis of empirical findings and theoretical assumptions. The advantage of the suggested validity approach is that it helps distinguish the roles of different component skills for different domains by determining whether they are related to fast and correct task solution processes or not. We conclude that not only ability score differences should be focused on when investigating the validity of the construct interpretation of ability scores, but also differences in the speed-ability relation and how they are affected by component skills considered relevant for the construct.

Interpreting the speed-ability relation in terms of construct-related response processes requires ruling out alternative explanations. For instance, test-wiseness could explain the speed-ability relation, as greater test-wiseness presumably makes test-takers both faster and more successful. However, one would assume that test-wiseness has a consistent impact across domains. This is obviously not the case given the differences between reasoning and reading in the relation between speed and ability. Moreover, the pattern of interaction effects for construct-related component skills speaks against this assumption as well.

Unpredicted but interestingly, the relation between reading speed and reading ability was also moderated by perceptual speed, but in a negative direction. This does not contradict our hypothesis, which was that perceptual speed does *not* moderate the speed-ability relation in a *positive* direction. The negative interaction effect does not mean that perceptual speed is involved in performing reading processes in the automated mode (what would have been supported by a positive moderation effect, as was the case for word meaning activation). It rather indicates that higher perceptual speed is associated with higher ability for persons working more slowly. Perceptual speed could have functioned as a resource, for instance to compensate for non-automatized processes. Word identification during reading can be based on different processes (cf. Perfetti, 2007): on extracting word meanings or decoding single letters. While word meaning activation might matter for the first process, perceptual speed might matter for decoding when the meaning cannot be directly retrieved. Higher perceptual speed might thus increase the probability of correct item solution when respondents invest time into decoding single words in order to understand the text as well as possible.

What do the results mean for the pursued research questions for reading? The results first indicate that solving PIAAC literacy items is rooted in typical reading-specific processes like word meaning activation. Word meaning activation was not only predictive of reading ability (main effect) but was also related to fast and correct solutions and thus involved in the response process (interaction effect). Secondly, higher perceptual speed, a component skill of reasoning, predicted reading ability (main effect). The unexpected negative interaction effect indicated that the speed-ability relation differed depending on a persons’ perceptual speed. The results suggested that perceptual speed was not related to automated cognitive processes but may, rather, represent a resource for compensating behavior. This result is highly interesting because it indicates that perceptual speed plays a different role in PIAAC literacy items than reading-specific component skills. Thus, examining the relations between component skills and speed-ability relations in tasks from a given domain can reveal whether and how component skills are involved in the solution process for complex tasks like reading.

The results for reasoning need to be interpreted in light of the general reasoning speed factor we modeled. Correlations between items were allowed in order to achieve an acceptable model fit for the measurement model. Correlations between easy items and between hard items were necessary, but not between items with a medium level of difficulty. This indicates that speed in reasoning tasks can be multidimensional, perhaps because strategies might change from easy to hard items. In this study, the latent reasoning speed factor is dominated by the speed respondents exhibited on items of medium difficulty. As a consequence, the results should primarily be interpreted in this respect, that is, perceptual speed might particularly play a role for items of medium difficulty. Other component skills might potentially be involved in easier or harder items. Hence, it would be interesting to investigate in future studies what other component skills are crucial in reasoning items depending on the level of item difficulty.

Regarding the research question on interaction effects, it can be concluded for reasoning that the component skill of perceptual speed seems to be important for reasoning tasks at least at a medium level of difficulty, while word meaning activation as is not related to automated processes when solving reasoning items.

### Limitations of the Study

We applied our validation approach to a subsample of the German PIAAC sample that was re-assessed within a longitudinal setting. This longitudinal setting was highly advantageous because it meant that the same individuals completed reading tasks, reasoning tasks, and both component skills tasks (i.e., word meaning activation and perceptual speed). However, there were also some limitations. First, each construct was only operationalized with one measure, which means that the latent variables we obtained may also reflect properties of the measure (e.g., the ability to deal with numerical material in the case of reasoning). Second, the selection of domain-specific component skills was limited. For instance, in the case of literacy, the component skill semantic integration (Richter and Naumann, 2009) would have been another candidate to affect the speed-ability relation. Third, the PIAAC study is a low-stakes assessment study, and test-takers’ motivation might have varied. Low motivation elicits cognitive processes that may be unrelated to the task solution. By contrast, the described approach is based on the assumption that speed in an item can be interpreted as the duration of a person’s cognitive processes and differences in the speed-ability relation as an indicator for differences in cognitive processing. Fourth, test-takers in the PIAAC-L assessment completed the PIAAC reading items in both 2012 and 2015. Although there was some time in between, test-takers might have gotten used to these kinds of tasks and have remembered seeing the same items 3 years ago. Thus, carry-over effects could have affected the results.

### Strengths and Limitations of the Validation Approach

In our view, the strength of the proposed validation approach is that information from the response process is used to support the validity of construct interpretations. Relations between component skills and the two ability tests (cf. main effects) did not reveal any differences between reading and reasoning, because word meaning activation and perceptual speed were positively predictive for both constructs. Only when considering component skills as a moderator of the relation between speed and ability were differences revealed between the reading and the reasoning tests. In the reasoning test, the relation between reasoning speed and reasoning ability was more positive for persons with higher perceptual speed. In the reading test, the relation between reading speed and reading ability was more negative for persons with higher perceptual speed. Hence, higher perceptual speed supports a higher degree of automatization in reasoning but plays a different role in reading – one possible explanation being to compensate for non-automated processes. However, these different roles only become visible when examining not just the relations between component skills and the product of task completion (relation between component skill and ability), but also the process of task completion (relation between component skill and the speed-ability relation). Although the data analysis is based on regression analyses with speed ‘predicting’ ability, we assume no causal direction in the relation between speed and ability. However, we do assume that differences in component skills can indeed ‘cause’ a different relation of speed and ability.

We assume that this approach is especially useful for validating the construct interpretation of constructs for which no single process model exists. A number of different processes are involved in tasks like reading and reasoning, making it challenging to use process information for validation (Kane and Mislevy, 2017, p. 11). Research on the reading process indicates a web of complex, entangled processes that are both top-down and bottom-up, both controlled and automated. The described approach only explicitly requires assumptions to be made about the involved component skills. However, it also implicitly assumes constant task-related cognitive processes. For assessments in which items are heterogeneous, the role of various component skills may vary across items, and different ones may even be required for different items. In such cases, it seems reasonable to investigate moderation effects at the item level as well.

An additional advantage of our approach is that it helps to collect not only convergent but also discriminant validity evidence. If we had only focused on sources of convergent validity, positive interaction effects could have also been caused by other factors such as restricted variance (cf. Cortina et al., 2018). When a certain level of a component skill that is also related to the target ability is considered, the variance in the target ability is restricted. Positive interactions might stem from the fact that the predictor restricts variance in the criteria. Thus, the analyses of discriminant sources provided additional support by showing that although the predictors and criteria were related, the positive interaction effects did not occur in all cases, but only for the hypothesized effects based on the theoretical assumptions. This supports the notion that the positive interaction effects for sources of convergent validity evidence are not the result of variance restriction. In addition, the collection of convergent and discriminant validity evidence, by referring to the same component skill for different constructs, allows conclusions about the distinctness or relatedness of two constructs in terms of their underlying processes. Two constructs differ in their underlying processes when a component skill positively moderates the speed-ability relationship of construct A but not of construct B. In the present study, perceptual speed as an underlying skill in terms of automation moderated the speed-ability relation for reasoning, but not for reading competence, and word meaning activation for reading competence, but not for reasoning. In future studies focused on constructs that are less divergent than reading and reasoning, where a component skill of one target ability cannot provide discriminant validity evidence for the other construct, one could also include component skills that can serve as sources of discriminant validity evidence for both constructs.

Furthermore, this approach is based on the assumption that sub-processes of the task solution process can be performed in an automatized mode. Hence, this approach is limited to constructs for which the dual processing framework holds. We assume that our approach is especially applicable to assessments from educational studies (e.g., PIAAC; OECD, 2016), which focus on assessing broad competence domains that require the interplay of various component skills. In any event, a sound theoretical basis concerning the involved component skills is required to derive hypotheses. However, it is conceivable that also variables apart from component skills can be used as moderating variables. Different behavior associated with different speed-ability relations could be originate from tasks that allow different solution strategies with one way being superior to the other, or by differently experienced participants (e.g., test-wiseness; Millman et al., 1965). In both cases, persons who worked faster and used superior solution strategies or had a higher test experience compared to other persons should have even higher scores.

Finally, although our validation approach aims to investigate the response process for validation purposes, it does not capture the intra-individual cognitive information processing for a single person completing a single item. Instead, our validation argument relies on statistical parameters describing relations across persons to infer meaningful characteristics of the response process.

### Future Directions

In general, the presented empirical findings can be seen as preliminary and must be supported by future studies. In this study, test score validation was challenged (cf. Kane, 2013) with alternative theories by collecting discriminant validity evidence. However, there was no cross validation checking, for instance. In the future, the proposed validation approach must prove itself with respect to other samples, other constructs and other component skills.

As previously argued, the validation approach is assumed to be especially useful when no single process model exists. From this, it follows that more than one component skill is likely to be involved in task solution in such cases. Hence, more than one component skill may moderate the speed-ability relationship and should be considered when collecting convergent validity evidence. The simultaneously inclusion of multiple, interrelated component skills (e.g., semantic integration and word meaning activation in the case of reading) in the model would affect the interpretation of effects. In this case, the effects would be estimated as partial regression coefficients controlling for the other predictors in the model.

As mentioned above, the role of component skills may vary across items depending on item characteristics. Moderation effects could also be tested at the item level by adding effects of (residual) response time on (residual) response within items (cf. Bolsinova et al., 2017b). Investigating how component skills moderate these item-specific effects would shed light on how the effect of being faster than expected on task success depends on certain component skills. The variation in the moderating effect across items could be related to item difficulty as well as item characteristics that determine it. Thus, our approach could be adapted to the item level in order to provide further insights into the response process at this level.

## Conclusion

Overall, this study proposed a novel validation approach that allows for investigating the role of component skills in response processes by focusing not only on the main effect of component skills on the targeted ability dimension, but also on how they influence the relation between speed and ability. As shown in the empirical example, including speed and interacting variables in the construct validation allows for testing specific hypotheses about the role of component skills in the task completion process, beyond their role in the task outcome.

## Ethics Statement

This study is based on data from the PIAAC-L study (GESIS – Leibniz Institute for the Social Sciences, German Socio-Economic Panel (SOEP) at DIW Berlin and LIFBI – Leibniz Institute for Educational Trajectories, 2017). As explicated by the Technical Report of PIAAC-L “the PIAAC-L Consortium is committed to adhering to survey ethics and undertaking any appropriate and effective measures required by data privacy concerns. These issues were already addressed by the international PIAAC project, and GESIS developed an elaborate data confidentiality strategy for the German PIAAC 2012 Scientific Use File” (Zabal et al., 2016, p. 26).

## Author Contributions

LE and FG contributed to the conception and design of the study. LE organized the database. LE and FG performed the statistical analysis. LE wrote the first draft of the manuscript. LE and FG contributed to revision, read, and approved the final version of the manuscript for submission.

## Conflict of Interest Statement

The authors declare that the research was conducted in the absence of any commercial or financial relationships that could be construed as a potential conflict of interest.

## Footnotes

1 For example items, see www.oecd.org/skills/piaac/Literacy%20Sample%20Items.pdf (accessed October 26, 2018).

2 For an example, see p. 10 https://www.diw.de/documents/publikationen/73/diw_01.c.570984.de/diw_ssp0339.pdf (accessed October 26, 2018).

3 For an example, see p. 11 https://www.diw.de/documents/publikationen/73/diw_01.c.570984.de/diw_ssp0339.pdf (accessed October 26, 2018).

## Appendix

**Table A1:** Measurement models reading (standardized results).

	Reading ability		Reading speed	
	Loading (SE)	Threshold (SE)	Loading (SE)	Intercept (SE)
Item 1	0.44 (0.09)	–1.47 (0.10)	0.63 (0.03)	20.48 (0.66)
Item 2	0.71 (0.05)	–1.04 (0.07)	0.60 (0.03)	22.59 (0.73)
Item 3	0.64 (0.06)	–0.98 (0.07)	0.51 (0.04)	16.73 (0.56)
Item 4	0.48 (0.06)	–0.67 (0.06)	0.56 (0.03)	27.89 (0.92)
Item 5	0.71 (0.03)	–0.65 (0.04)	0.71 (0.02)	18.61 (0.42)
Item 6	0.57 (0.04)	–0.02 (0.04)	0.56 (0.02)	19.11 (0.45)
Item 7	0.59 (0.04)	–1.07 (0.05)	0.64 (0.02)	17.16 (0.39)
Item 8	0.45 (0.04)	0.07 (0.04)	0.66 (0.02)	21.13 (0.50)
Item 9	0.64 (0.04)	–0.57 (0.04)	0.61 (0.02)	22.57 (0.53)
Item 10	0.55 (0.04)	0.10 (0.04)	0.54 (0.03)	20.13 (0.46)
Item 11	0.82 (0.02)	–0.29 (0.04)	0.69 (0.02)	18.62 (0.44)
Item 12	0.49 (0.04)	0.67 (0.04)	0.63 (0.02)	17.51 (0.41)
Item 13	0.52 (0.04)	0.07 (0.04)	0.52 (0.03)	18.83 (0.43)
Item 14	0.64 (0.05)	–0.47 (0.06)	0.60 (0.03)	17.06 (0.55)
Item 15	0.50 (0.05)	–0.15 (0.05)	0.62 (0.03)	20.86 (0.68)
Item 16	0.52 (0.05)	0.25 (0.05)	0.49 (0.04)	17.05 (0.57)
Item 17	0.60 (0.06)	0.71 (0.06)	0.55 (0.04)	18.18 (0.60)
Item 18	0.41 (0.06)	0.22 (0.06)	0.71 (0.03)	21.11 (0.70)
Item 19	0.63 (0.07)	–0.98 (0.09)	0.46 (0.05)	16.26 (0.70)
Item 20	0.73 (0.07)	–1.65 (0.11)	0.50 (0.05)	19.07 (0.81)
Item 21	0.62 (0.12)	–2.32 (0.20)	0.55 (0.04)	20.83 (0.85)
Item 22	0.70 (0.05)	–0.31 (0.07)	0.62 (0.04)	19.97 (0.80)
Item 23	0.46 (0.07)	–0.55 (0.08)	0.49 (0.05)	16.64 (0.71)
Item 24	0.51 (0.08)	–0.67 (0.08)	0.53 (0.04)	19.26 (0.83)
Item 25	0.63 (0.04)	–0.54 (0.05)	0.75 (0.02)	23.75 (0.63)
Item 26	0.37 (0.05)	0.30 (0.05)	0.63 (0.03)	18.30 (0.51)
Item 27	0.58 (0.04)	–0.15 (0.05)	0.69 (0.02)	21.04 (0.55)
Item 28	0.49 (0.05)	–0.86 (0.05)	0.49 (0.03)	16.25 (0.45)
Item 29	0.74 (0.04)	–0.94 (0.06)	0.52 (0.03)	17.40 (0.48)
Item 30	0.79 (0.04)	–0.50 (0.06)	0.64 (0.03)	21.68 (0.75)
Item 31	0.70 (0.05)	–0.32 (0.06)	0.60 (0.03)	15.72 (0.56)
Item 32	0.67 (0.05)	–0.30 (0.06)	0.62 (0.03)	16.77 (0.58)
Item 33	0.64 (0.05)	–0.89 (0.06)	0.61 (0.03)	20.59 (0.56)
Item 34	0.62 (0.04)	–0.07 (0.04)	0.54 (0.03)	17.34 (0.48)
Item 35	0.64 (0.04)	–0.52 (0.05)	0.59 (0.03)	19.52 (0.52)
Item 36	0.57 (0.06)	–0.16 (0.06)	0.49 (0.04)	16.97 (0.66)
Item 37	0.71 (0.06)	0.46 (0.06)	0.56 (0.04)	17.75 (0.70)
Item 38	0.75 (0.05)	–0.47 (0.07)	0.47 (0.05)	19.14 (0.75)
Item 39	0.58 (0.06)	–1.47 (0.08)	0.63 (0.02)	15.95 (0.42)
Item 40	0.42 (0.05)	0.45 (0.05)	0.56 (0.03)	19.16 (0.51)
Item 41	0.74 (0.04)	0.33 (0.04)	0.58 (0.03)	20.26 (0.55)
Item 42	0.77 (0.03)	0.33 (0.04)	0.56 (0.03)	23.43 (0.64)
Item 43	0.58 (0.04)	–0.36 (0.05)	0.46 (0.03)	16.51 (0.45)
Item 44	0.77 (0.04)	–0.31 (0.06)	0.65 (0.03)	20.35 (0.72)
Item 45	0.66 (0.05)	–0.08 (0.06)	0.64 (0.03)	23.06 (0.82)
Item 46	0.56 (0.07)	0.74 (0.06)	0.72 (0.03)	18.51 (0.67)
Item 47	0.50 (0.07)	0.85 (0.07)	0.55 (0.04)	18.24 (0.67)
Item 48	0.49 (0.06)	0.35 (0.06)	0.69 (0.03)	17.93 (0.64)
Item 49	0.68 (0.07)	1.21 (0.07)	0.57 (0.04)	17.69 (0.65)

**Table A2:** Measurement models reasoning (standardized results).

	Reasoning ability		Reasoning speed	
	**Loading (SE)**	**Threshold (SE)**	**Loading (SE)**	**Intercept (SE)**
Item 1	0.87 (0.05)	–2.95 (0.28)	0.36 (0.03)	4.01 (0.10)
Item 2	0.77 (0.10)	–2.61 (0.18)	0.47 (0.03)	4.33 (0.11)
Item 3	0.56 (0.07)	–1.64 (0.08)	0.55 (0.03)	4.79 (0.12)
Item 4	0.77 (0.07)	–2.41 (0.14)	0.59 (0.03)	4.11 (0.10)
Item 5	0.60 (0.05)	–1.24 (0.06)	0.62 (0.02)	4.58 (0.11)
Item 6	0.58 (0.04)	–0.46 (0.04)	0.66 (0.02)	5.17 (0.13)
Item 7	0.70 (0.09)	–2.05 (0.11)	0.66 (0.02)	4.40 (0.11)
Item 8	0.66 (0.05)	–0.94 (0.05)	0.68 (0.02)	4.93 (0.12)
Item 9	0.72 (0.04)	–0.73 (0.05)	0.66 (0.02)	4.86 (0.12)
Item 10	0.52 (0.04)	–0.37 (0.04)	0.43 (0.03)	4.79 (0.12)
Item 11	0.60 (0.04)	–0.37 (0.04)	0.44 (0.03)	4.44 (0.11)
Item 12	0.77 (0.03)	0.00 (0.04)	0.50 (0.03)	4.82 (0.12)
Item 13	0.86 (0.03)	–0.29 (0.04)	0.48 (0.03)	5.03 (0.13)
Item 14	0.71 (0.03)	–0.31 (0.04)	0.37 (0.03)	4.67 (0.12)
Item 15	0.70 (0.04)	0.60 (0.05)	0.16 (0.04)	5.42 (0.14)

**Table A3:** Measurement models perceptual speed and word meaning activation (standardized results).

	Loading (SE)	Intercept (SE)
Perceptual speed		
Interval 1 (0–30 s)	0.85 (0.01)	3.09 (0.06)
Interval 2 (30–60 s)	0.89 (0.01)	4.23 (0.08)
Interval 3 (60–90 s)	0.82 (0.01)	4.45 (0.09)
Word meaning activation		
Number of correct answers	0.87 (0.02)	0.00 (0.03)

**Table A4:** Residual correlations (and standard errors) in the measurement model for reasoning speed (standardized results).

	Item1	Item2	Item3	Item4	Item5	Item 6	Item 7	Item 8	Item 9	Item10	Item11	Item12	Item13	Item14
Item1														
Item2	0.25 (0.03)													
Item3	0.18 (0.03)	0.28 (0.03)												
Item4	0.19 (0.03)	0.35 (0.03)	0.28 (0.03)											
Item 5														
Item 6														
Item 7														
Item 8														
Item 9														
Item10														
Item11										0.24 (0.03)				
Item12										0.32 (0.03)	0.29 (0.03)			
Item13										0.32 (0.03)	0.38 (0.03)	0.41 (0.03)		
Item14										0.34 (0.03)	0.39 (0.03)	0.39 (0.03)	0.55 (0.03)	
Item15										0.41 (0.03)	0.41 (0.03)	0.38 (0.03)	0.44 (0.03)	0.48 (0.03)
